# Health impact assessment of particulate pollution in Tallinn using fine spatial resolution and modeling techniques

**DOI:** 10.1186/1476-069X-8-7

**Published:** 2009-03-03

**Authors:** Hans Orru, Erik Teinemaa, Taavi Lai, Tanel Tamm, Marko Kaasik, Veljo Kimmel, Kati Kangur, Eda Merisalu, Bertil Forsberg

**Affiliations:** 1 Department of Public Health, University of Tartu, Ravila 19, Tartu 50411, Estonia; 2 Department of Public Health and Clinical Medicine, Umea University, Umea SE-901 87, Sweden; 3 Estonian Environmental Research Centre, Marja 4d, Tallinn 10617, Estonia; 4 Department of Physics, University of Tartu, Riia 142, Tartu 50414, Estonia; 5 Department of Ecology and Geography, University of Tartu, Vanemuise 46, Tartu 50414, Estonia; 6 Institute of Agricultural and Environmental Sciences, Estonian University of Life Sciences, Kreutzwaldi 64, Tartu 51014, Estonia; 7 Department of Geography, King's College London, Strand, London ,WC2R 2LS, UK

## Abstract

**Background:**

Health impact assessments (HIA) use information on exposure, baseline mortality/morbidity and exposure-response functions from epidemiological studies in order to quantify the health impacts of existing situations and/or alternative scenarios. The aim of this study was to improve HIA methods for air pollution studies in situations where exposures can be estimated using GIS with high spatial resolution and dispersion modeling approaches.

**Methods:**

Tallinn was divided into 84 sections according to neighborhoods, with a total population of approx. 390 000 persons. Actual baseline rates for total mortality and hospitalization with cardiovascular and respiratory diagnosis were identified. The exposure to fine particles (PM_2.5_) from local emissions was defined as the modeled annual levels. The model validation and morbidity assessment were based on 2006 PM_10 _or PM_2.5 _levels at 3 monitoring stations. The exposure-response coefficients used were for total mortality 6.2% (95% CI 1.6–11%) per 10 μg/m^3 ^increase of annual mean PM_2.5 _concentration and for the assessment of respiratory and cardiovascular hospitalizations 1.14% (95% CI 0.62–1.67%) and 0.73% (95% CI 0.47–0.93%) per 10 μg/m^3 ^increase of PM_10_. The direct costs related to morbidity were calculated according to hospital treatment expenses in 2005 and the cost of premature deaths using the concept of Value of Life Year (VOLY).

**Results:**

The annual population-weighted-modeled exposure to locally emitted PM_2.5 _in Tallinn was 11.6 μg/m^3^. Our analysis showed that it corresponds to 296 (95% CI 76528) premature deaths resulting in 3859 (95% CI 10236636) Years of Life Lost (YLL) per year. The average decrease in life-expectancy at birth per resident of Tallinn was estimated to be 0.64 (95% CI 0.17–1.10) years. While in the polluted city centre this may reach 1.17 years, in the least polluted neighborhoods it remains between 0.1 and 0.3 years. When dividing the YLL by the number of premature deaths, the decrease in life expectancy among the actual cases is around 13 years. As for the morbidity, the short-term effects of air pollution were estimated to result in an additional 71 (95% CI 43–104) respiratory and 204 (95% CI 131–260) cardiovascular hospitalizations per year. The biggest external costs are related to the long-term effects on mortality: this is on average €150 (95% CI 40–260) million annually. In comparison, the costs of short-term air-pollution driven hospitalizations are small €0.3 (95% CI 0.2–0.4) million.

**Conclusion:**

Sectioning the city for analysis and using GIS systems can help to improve the accuracy of air pollution health impact estimations, especially in study areas with poor air pollution monitoring data but available dispersion models.

## Background

Health impact assessment (HIA) is a combination of procedures, methods and tools by which a policy, programme or project may be evaluated based on its potential effects on the health of a population, and the distribution of those effects [1]. Knowledge of the exposure, baseline mortality or morbidity in the population as well as exposure-response functions from epidemiological studies helps us to estimate trends in negative health effects associated with alternative scenarios.

One of the first important air pollution HIA was conducted by Künzli et al. [2]. This study estimated the impact of traffic particulate pollutants in Austria, France and Switzerland which were found to cause 40 000 premature deaths, 25 000 new cases of chronic bronchitis, and many chronic bronchitis episodes and asthma attacks. Other early HIA reports found that men in Holland and the US population as a whole lose 1–3 years of life due to air pollution [3,4].

Recommendations for HIAs of environmental factors had been published by the World Health Organisation (WHO) and European Centre for Environment and Health [5,6]. Even though several authors [7,8] have subsequently discussed some of the difficulties associated with HIAs in this field, the basic principles have remained unchanged.

A recent large detailed outdoor air pollution HIA was carried out by the Apheis Project which covered 23 European cities [9]. The influence of fine particles (PM_2.5_) on health was assessed by the number of premature deaths and Years of Life Lost (YLL). This study showed that a reduction of PM_2.5 _(particulate matter with diameter less than 2.5 μm) concentration to 15 μg/m^3 ^in these cities could possibly avert almost 17 000 premature deaths. The average life expectancy at birth would increase more than 2 years in heavily polluted cities like Bucharest, Rome, Tel Aviv [9]. If the WHO air quality guidelines (PM_2.5 _annually < 10 μg/m^3^) were followed in these cities, the premature death rate would be reduced by 41/100 000 [10].

In a WHO report, the average life expectancy at birth among all European Union (EU) citizens in 2000 was estimated to be shortened by 8.6 months due to fine particles and PM_2.5 _levels were thought to cause the premature death annually of 348 000 people in Europe [11]. Globally, the annual number of premature deaths due to outdoor fine particles is considered to be at least 800 000 [12].

If the currently and previously agreed policies related to the emission reductions of PM were fulfilled, the average life expectancy in Europe would increase by 2.3 months by 2020 [13]. This is equal to 80 000 premature deaths and more than one million YLL avoided annually.

Golub & Strukova [14] have analyzed numerous HIAs in Russia and found that air pollution causes 87 000 deaths annually in the Russian Federation, and this comprises ~4% of the total mortality. Yorifuji et al. have observed that if annual PM_2.5 _level in Tokyo, Japan were lowered to below 12 μg/m^3 ^in 2005, total mortality would decrease 8% and 6 700 premature deaths would be avoided [15]. A Swedish study (2005) on the impact of particulate matter established that it could cause annually more than 4700 premature deaths in cities and almost 600 in the countryside of Sweden [16].

The economic costs of health loss due to outdoor pollution can be estimated as well. In the EU, the external costs of air pollution are estimated to be 50–161 billion Euros annually due to premature mortality and 29 billion € from morbidity. This represents more than 1% of the Union's GDP in 2005 [13]. It is also important to note that the majority of the morbidity-related external costs from air pollution are related to the public health sector and not to the health care sector [17].

Even though several indicators have been used for HIAs, the main goal is to quantify the negative effects of risk factors and provide guidelines for policymakers, developers, planners, etc., to assist them in the mitigation of negative health effects by decreasing exposure to air pollution.

### Tallinn

The sources of air pollution in Estonian capital Tallinn (~390 000 inhabitants) are quite complex with an important role played by local heating. Thus, the health impacts of the air pollution are best characterized using PM_2.5 _using relative risk assumptions from studies of a mix sources. The negative effects of chronic exposure to fine particles, even at low concentrations, has previously been shown in various epidemiological studies [18,19].

The aim of this study was to estimate the added local health impacts due to emissions in Tallinn. HIAs in such towns as Tallinn help to quantify the health effects of air pollution in less polluted average-size cities and in less studied regions where economic transitions have been very rapid. The current study improves the explanatory power of HIA methods by incorporating modeling and sectioning approaches for cities where a air quality measuring network is rare or absent.

## Methods

In the current HIA study, data on population, baseline mortality and morbidity, air pollution exposure, exposure-response functions, socio-economical condition and health-care expenses were gathered and analyzed.

### Baseline population, mortality and morbidity data

**Figure 1:**
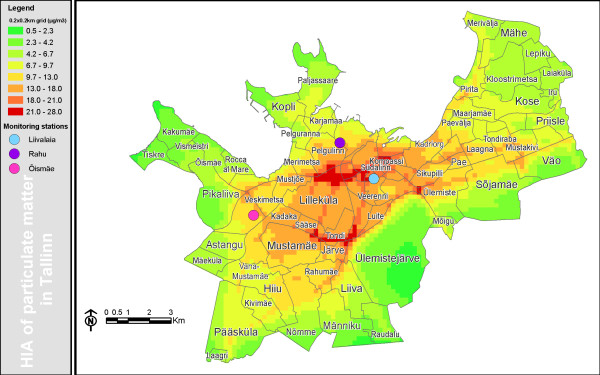
**Modeled (200 × 200 m grid) annual average concentration of PM_2.5 _in Tallinn, μg/m^3^**.

Population data for Tallinn is based on the Population Register (02.02.2007) according to address and registration in the following age groups: 0–6, 7–17, 18–27, 28–37, 38–47, 48–57, 58–67, 68+ years. The citizens' residences were divided into sections according to neighborhoods (regions with similar geographical, socio-economic, etc., patterns), forming small administrative units (smaller than city districts) used in city planning and management. Altogether 84 sections (Fig 1, 3 and 4) were formed in order to identify site-specific exposure to air pollution and identify the areas with greatest risk. A 'neighborhood' is considered to be a small and homogeneous section, where air pollution as a risk factor is assumed to be similar. The age-structure of the population in each section was identified and used for calculation of YLL with life-tables methodology. Each section also belongs to one of the 8 city districts (Table 1).

**Table 1:** The number of premature death due to PM_2.5 _pollution in Tallinn

City district	Number of population	Annual exposure to local PM_2.5 _(μg/m^3^)	Number of premature deaths (95% CI)	Number of premature deaths 1/1000 (95% CI)	The loss of life expectancy in years (95% CI)
Haabersti	38 031	9.5	23 (6–42)	0.60 (0.16–1.10)	0.52 (0.14–0.90)

Mustamäe	62 589	14.0	63 (16–112)	1.01 (0.26–1.79)	0.78 (0.20–1.34)

Nõmme	38 268	7.2	18 (5–31)	0.47 (0.13–0.81)	0.40 (0.11–0.68)

Kesklinn	47 105	17.1	51 (13–91)	1.08 (0.28–1.93)	0.94 (0.25–1.62)

Kristiine	28 878	16.2	30 (8–54)	1.04(0.28–1.87)	0.89 (0.24–1.53

Lasnamäe	107 280	10.2	73 (19–131)	0.68 (0.18–1.22)	0.56 (0.15–0.97)

Pirita	13 192	6.4	5 (1–8)	0.38 (0.08–0.61)	0.36 (0.09–0.61)

Põhja-Tallinn	53 621	9.3	33 (9–59)	0.62 (0.17–1.10)	0.52 (0.14–0.89)

**Total**	**388 964**	**11.6**	**296 (76–528)**	**0.76 (0.20–1.36)**	**0.64 (0.17–1.10)**

The total regional baseline mortality was retrieved from statistics on Estonia (International Classification of Diseases – ICD-10, A00-Y98). The morbidity calculations were carried out using hospitalization data from the Estonian Health Insurance Fund (EHIF), which covers the whole population and is the sole purchaser of health care services in the country.

Hospitalizations due to two main disease groups were included in the calculations: cardiovascular (I00-I99) and respiratory causes (J00-J99). Cardiac admissions (I20-I25) and cerebrovascular admissions (I60-I69) were also used for the exposure-response work on cardiovascular hospitalizations. The short-term effects of high pollution levels on mortality were not calculated separately as according to several authors [2,9,16] these are already included in exposure-response function of long-term mortality.

### Exposure assessment

The annual levels of locally-emitted PM_2.5_, as well as PM_10 _for model validation were estimated using model AirViro [20], based on emission data for traffic, industry, local and central heating along with meteorological parameters with grid resolution 200 × 200 meters. A database of local heating emissions was developed during the current study, using a previous questionnaire study on fuel consumption results [21] and the European Environmental Agency's emission factors for small combustion devices based fuel consumption. The traffic flows have been measured in Tallinn in 2005 and 2006. The emission factors were taken from CORINAIR [22] for traffic and from a database of pollution licenses for industry, central heating etc. The temporal resolution for traffic was one hour, a working day for industrial sources and one month for boiler houses and residential heating.

For model validation the PM_2.5 _and PM_10 _modeled levels were compared with air quality monitoring data from Rahu, Õismäe and Liivalaia receptor points (Fig. 1) for three meteorological years, 2004–2006. The Liivalaia monitoring station is located in the city centre and it represents a typical city hotspot. The Õismäe monitoring station is an urban background station located in a typical city region with apartment houses where a large number of the Tallinn population reside. The Rahu monitoring station is located in a region of residential houses together with a nearby railway with a number of the diesel trains passing by on a daily basis. In each monitoring station, the concentration of PM_10 _is routinely measured by beta-attenuation analyzers (Thermo Andersen FH-62). In the Õismäe station, the PM_10 _levels are measured by the reference method (Digitel DHA-80) as well. The PM_2.5 _level is monitored only at the Õismäe station, and this is done by beta-attenuation analyzer (Thermo Andersen FH-62).

The annual levels of PM_2.5 _were calculated for all 84 Tallinn sections using the average concentration of modeled grid cells in a section. The average concentration for each section was then assigned to all residents of that neighborhood. Only individuals of age 28+ were included in analyses, as the US cohort study [23].

Short-term effects of air pollution were calculated using mean daily average concentrations of PM_10 _from the 3 monitoring stations in 2006 and morbidity data for all age-groups. For each neighborhood the most representative station was used based on the location.

### Exposure-response functions, calculation of mortality and morbidity

To describe the long-term effect of air pollution on mortality, the broadly employed US ACS study relative risk RR = 1.06 (95% CI 1.02–1.11) per 10 μg/m^3 ^increase of PM_2.5 _was used as the exposure-response relationship [23]. For calculations of respiratory hospitalizations due to short-term air pollution episodes, RR = 1.0114 (95% CI 1.0062–1.0167) per 10 μg/m^3 ^increase of PM_10 _was used [24]. For cardiovascular hospitalizations we used a weighted average RR = 1.0073 (95% CI 1.0047–1.0093) per 10 μg/m^3 ^increase of PM_10 _based on the effect on cardiac and cerebrovascular admissions from a COMEAP meta-analysis [25].

The cases (mortality and morbidity) were calculated in absolute and relative numbers for all sections in Tallinn. The following equation was used:

Δ*Y *= (*Y*_0 _× *pop*) × (*e*^*β *× *X *^- 1)

where *Y*_0 _is the baseline rate; *pop *the number of exposed persons; *β *the exposure-response function (relative risk) and *X *the estimated excess exposure.

The number of YLL was calculated using "life-tables" methodology, where the hypothetical life expectancy is compared with the life expectancy affected by air pollution. The calculation of YLL and changes in life expectancy were facilitated by a WHO Centre for Environment and Health developed program AirQ 2.2.3 (Air Quality Health Impact Assessment Tool) [26]. For calculation of hospitalizations, the short-term effects module of AirQ was used. The number of hospitalization cases was determined at different exposure intervals (10–19.9; 20–29.9; ...); no effect was assumed below 10 μg/m^3^.

### Assessment of socio-economic external costs

Air pollution affects economic and social well-being through mortality and morbidity. Morbidity, in turn, affects the health and productivity of the labor force. In this study, the direct costs related to morbidity were calculated using costs of hospitalization, salary compensation during sick leave and loss of labor input (based on GDP per capita). The data for hospitalization cost calculations were provided by EHIF, where the average costs of a hospitalization case due to respiratory disease or general internal disease in 2005 was €1239 and €778 respectively [27]. The same source was used to identify the time spent in hospital (6.9 days) and the value of the average compensation of a workday (~€10).

For the country as a whole and its development prospects, the long-term outcomes and costs of air pollution effects are even more important than the direct costs. This means that in a case of premature death, people can lose decades of life-years but direct costs appear only in the actual year of death. The concept of *Statistical Value of Life *(SVL) and *Value Of Life Year *(VOLY) are used to express the cost of lost lives and life-years. These concepts stem from people's contribution to GDP, typical work time, salary and sometimes health care (compensation and decreased productivity) costs [28,29]. As there are no comprehensive statistical life valuation studies in Estonia, the conversion coefficient between GDP and the statistical value of life was derived from international meta-analyses (statistical value of life being on average equal to 120 times GDP *per capita *in a country) [30,28]. Value of a life year was calculated from the statistical value of life following the formula:

$$VOLY=\frac{SVL_{A}}{T-A}$$

where *VOLY *is statistical value of life year; *SVL *statistical value of life; *A *age when the case happened; *T *life expectancy; *T *- *A *loss of life.

A sensitivity analysis was performed using minimal and maximal economic statistical values of life and life-year to describe the range of potential errors.

## Results

### Baseline population, mortality and morbidity data

Altogether, 388 964 registered residents of Tallinn were identified in 84 sections of the city. Population-wise the biggest sections had more than 15 000 residents while some of the smallest had less than 100. The population density varied a great deal as well. In the majority of sections, the number of residents ranged from 3 000 to 16 000.

Based on mortality data, the mortality rates in different age groups were found (average 1 136 cases per 100 000 citizens per year) and the numbers were calculated in all 84 sections for the reference year 2006. The baseline hospitalization rates were determined separately for cardiovascular and respiratory admissions per 100 000 people using the same principles. The analysis showed 3 945 and 1 266 annual admission cases respectively per 100 000 people.

### Exposure levels

The city centre and nearby regions with local heating can be clearly differentiated as areas with higher exposure to fine particles (Fig. 1). The concentrations are also higher in other regions, especially those adjacent to the city centre. High concentrations also appear in smaller residential areas, particularly near busy streets (Fig. 1). The lowest concentrations were in the Tallinn fringe area with small but densely populated residential neighborhoods.

The exposure to fine particles was calculated by sections, using the modeled annual PM_2.5 _levels in 200 × 200 m grids in Tallinn. Modeling was based only on local sources as no data about background fine PM in Estonia are available. The background could be illustrated by the difference between modeled values (based on local sources) and measured values (local sources and regional background). Since we found that Tallinn itself contributes approx 0.4 μg/m^3 ^to the unknown regional background, it was subtracted from the modeled exposure values in order to calculate health impacts only associated with the levels above those outside the city.

**Figure 2:**
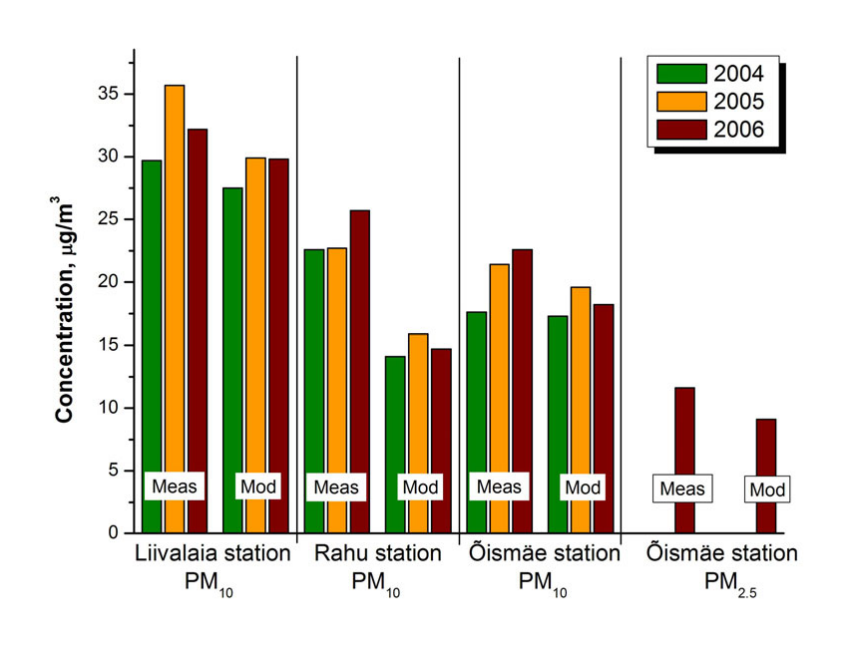
**Measured and modeled PM_10 _yearly average in monitoring stations**.

The difference between modeled and measured mean PM_2.5 _values in Õismäe station was 21% in 2006 (Fig. 2). The 2–3 μg/m^3 ^variation in PM_2.5 _annual values from the model indicates a somewhat lower background than expected. The average difference for all three monitoring stations above modeled PM_10 _levels over three years measurements was 18.8%. The biggest difference was in Rahu monitoring station, which is close to a railway with diesel locomotives and less known emissions, where measured and modeled PM_10 _values differed by 37%. At both Liivalaia and Õismäe monitoring stations the measured and modeled concentrations of PM_10 _differed by only 11% (Fig. 2). As agreements between the measured and modeled PM_10 _levels for the monitoring stations were fairly good, we have assumed that the model could also represent real particle levels fairly well at other receptor points in the city.

The concentration of particulate matter (PM_10_) at the 3 monitoring stations differed quite much. Generally, the concentration was the highest in the town centre and the lowest in the residential areas. This is expected, as the former has busy traffic and the latter is an apartment house area. It is noteworthy that in spring for some time the concentration at Õismäe station was surprising higher than in the centre town. As PM_2.5 _levels at that time were not very high, these high pollution episodes were presumably driven by coarse particles (PM_2.5–10_).

### Health impacts

**Figure 3:**
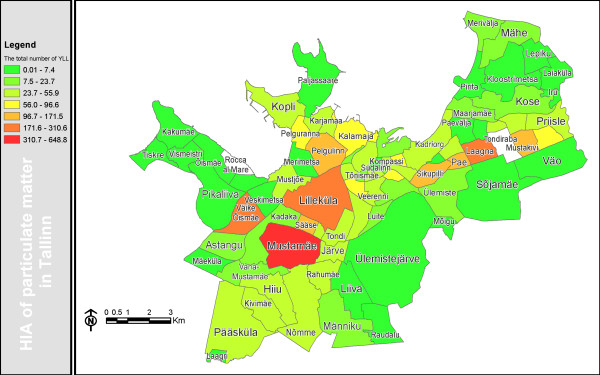
**The total number of YLL due to PM_2.5 _pollution in Tallinn**.

As some neighborhoods ('sections') had very few deaths, the estimated number of premature deaths attributed to the additional (local) particle pollution is presented at the level of city district (Table 1), whereas YLL is given at the level of neighborhood section (Fig. 3).

**Figure 4:**
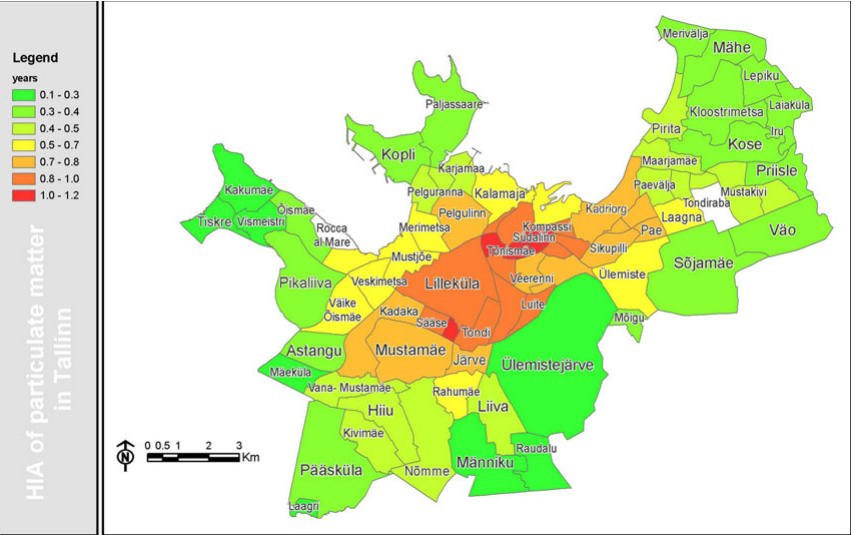
**Decrease of life-expectancy due to PM_2.5 _pollution in Tallinn**.

Our analysis shows that locally emitted air pollutants could be estimated to cause 296 (95% CI 76–528) premature deaths per year in Tallinn. According to the AirQ calculations using life tables these deaths correspond to 3 859 (95% CI 1 023–6 636) YLL, which is 988 YLL per 100 000 citizens. As a total number, the greatest loss (235–650 YLL) was in neighborhoods with a large number of citizens (25 000–50 000), e.g., Mustamäe, Lilleküla, Väike-Õismäe and Laagna (Fig. 3). The relative largest losses appeared in the city centre neighborhoods Kompassi, Südalinn, Tõnismäe.

Air pollution in Tallinn would then reduce the life-expectancy of the residents by on average 0.64 (95% CI 0.17–1.10) years, what corresponds to 7.7 months. The reduction is much greater in the city centre, e.g., in the Kompassi neighborhood where it reaches up to 1.17 years, whereas in the least polluted neighborhoods the decrease of life-expectancy remains between 0.1–0.3 years (Fig. 4). The average number of YLL per premature death is approx. 13 years.

Many of the negative health influences emerge in risk groups (people with respiratory and cardiovascular disease, elderly, etc.) [19]. Nevertheless, healthy people may be affected as well. Synergistic interactions with air pollution can appear to occur with other diseases.

Regarding morbidity, short-term exposure to PM_10 _is estimated to cause 71 (95% CI 43–104) respiratory hospitalizations per year in Tallinn. Even though RR is lower, the baseline frequency of cardiovascular hospital admissions is higher than for respiratory and the attributed absolute number is greater; 204 (95% CI 131–260) pollution related hospitalizations per year.

### Economic costs

There were 275 short-term air pollution related hospitalizations in Tallinn. Drawing from the average treatment cost data, the total direct cost to treat these air pollution related hospitalizations would be ~€245 000. For the days spent in the hospital ~€2 600 was paid to compensate temporary loss of income. The national economy lost input from hospitalized individuals to the value of €44 000 annually.

The statistical value of a life in Estonia in 2005 was estimated around 1 million € and the statistical value of a life-year ~€39 000. Based on these values the total indirect loss from air pollution caused premature deaths (296) adds up to €150 (95% CI 40–260) million.

In summary, the major external economic costs related to exposure to outdoor air pollution add up to €150.3 (95% CI 40.2–260.4) million. The majority stems from loss of life-years from premature deaths. This represents ~2.9% of Tallinn's GDP (€5.2 billion in 2005).

## Discussion

### Exposure assessment and benefits from methodological advances

While the methodology for HIA in this study follows generally accepted principles [31], major differences have been found in the exposure assessments. In the case of Tallinn, air pollution has been measured in only 3 monitoring sites. Thus, it was necessary to use dispersion modeling to gain an adequate level of detail for exposure assessment by city sections. The model validation showed fairly good agreement with the monitored levels, although the model generally underestimated the particle concentrations. The reasons for this may be the lack of a background concentration and an incomplete emission database; for example the high levels of PM measured at springtime because of road dust.

In our study, the sectioning was determined by a variety of natural and social factors, but the final results were found to follow to a large degree the municipal districts in Tallinn. Detailed population data was easily available even for small neighborhoods. In this case, it was feasible to employ this data in order to increase the accuracy of the analysis. Nevertheless, such detailed data is not always available. Population exposure is usually calculated as the average of monitored particulate matter in a large area subsequently adjusted for population density. We assume that in smaller cities with sparse monitoring networks, it would be more relevant to use GIS technologies for calculating average concentration by neighborhood and explicit demonstrating any variations. There might be contradictions with larger studies such as the one undertaken here, as the latter exposure-response coefficients are determined using different exposure assessment methods, but the differences would not be major.

The place of residence was used as the exposure position presuming that the greatest portion of the day is spent there. This is similar to other epidemiological studies from which exposure-response coefficients were taken. Furthermore, site of dwelling was the only data available from the population register. The amount of time a person spends in the residence area and outside of it (work, studies, etc.) affects individual exposure levels, however current methodology does not permit consideration of these variations. Neither could they be considered in the studies providing our exposure-response functions. When doing analysis with such accuracy (which is possible with modeling), individual factors such as a home's exact distance from the street, other pollution sources, individual sensibility to pollutants, etc. could play an important role.

As expected, the exposure was highest in the city centre and close to busy streets. People who live or work there, rather than the people who drive through, are more exposed to pollution. The number of people living in Tallinn is possibly greater than that indicated by the data used because of optional registration. In the sectioning process we also lost ~3% of persons, due to mismatching between Population and Land Register datasets.

### Critical issues

Firstly, the baseline population and health data are vital determinants affecting HIA results. Similar statements were made by Tainio et al. [32] in their statistical modeling study. In Tallinn, when looking at the absolute numbers, the highest number of casualties occurred in Lasnamäe, as the largest number of people lives in this neighborhood. If we look at relative values (e.g. YLL per 100 000), the greatest numbers are in the city centre and Kristine neighborhood. Thus, in Mustamäe and Lasnamäe the absolute number of casualties is similar as air pollution exposure is much higher in Mustamäe (Table 1). The age structure of the different populations also plays a role, but is quite minor.

Questions may arise about the possibility of over (under)estimation of the health impacts. The main basis for overestimation is the high baseline mortality rate (driven by external causes) in Estonia. This magnifies the relative exposure impact. If the health of the population is generally weak, the residents could likely be more sensitive to air pollution. In some cases, as in the Reshetin & Kazazyan study, where air pollution was said to cause 15–17% of mortality in Russia [33], the health impacts are probably overestimated because of very high base-line mortality (to a large extent related to alcohol consumption). Of course, we should not be too conservative in our estimations. In Helsinki, where the air pollution influence of busses was assessed, the results could be underestimated because only exhaust particles, which are seen as more toxic, were taken into account [34].

Secondly, the choice of a dose-response relationship represents an even more critical determinant of HIA results than any consideration of baseline data. For long-term mortality assessment, we applied the relative risk (Pope et al. [23]) from the ACS study that is broadly used in HIA studies. Since most of the stations in that survey were in urban surroundings, the combustion particles (prevailing in the city centre) could cause relative risk up to 1.17 per 10 μg/m^3 ^increase of PM_2.5_, as Jerret et al. found in the California cohort used in the ACS study [35]. Thus, we could have underestimated the effects on mortality. There are many other exposure-response coefficients available, but there is a great deal of differences of opinion, and additional studies are needed in order to determine which coefficient is the best.

A third problematic aspect lies in the choice of pollutant as the pollution indicator. We assumed that most of the health effects of air pollution could be quantified with PM_2.5_, especially due to the impact of local heating. In the HIA by Tonne et al. (2007), where effects of PM_2.5 _and NO_2 _from traffic were compared, there was a slightly greater influence on health recognized when NO_2 _was the indicator [36]. This means that we may have underestimated the pollution impact in the city centre. One possibility would be to conduct HIAs with several pollutants, so that alternative exposure rate scenarios could be designed. However, a simple addition of the effects of different pollutants would lead to overestimation and would be methodologically wrong.

The fourth critical issue is the exposure data in combination with any assumed threshold level of health effects. Studies have shown that fine particulate matter can cause negative effects on concentrations below current limit values [37]. In principle, we have assumed that the local contribution has the main impact. The background concentration is often used as the reference concentration. However, as the background is here presumed (as there is no study) to be quite low in Tallinn (2–3 μg/m^3^), we might have a slight over-estimation in our results, if a threshold level exists above this background. Our modeled PM levels seem to correspond to measured levels quite well except for one measuring site. For the short-term effects on hospital admissions, we calculated effects only above daily means of 10 μg/m^3^. If we had built this calculation on yearly mean values instead, and calculated the attributable cases above an estimated regional background (likely 4–6 μg/m^3^), we would have estimated a larger number of excess cases.

### Broader relevance of the results

Even though air pollution exposure in Tallinn is relatively low, the number of premature deaths and hospital admissions is rather high. As baseline cardiovascular hospitalization is known to be much higher in Estonia compared to respiratory hospitalization, the big differences between them was predictable. The negative effects on morbidity could also be found in family physician and other doctors' outpatient records. However, due to lack of relevant data, these morbidity cases were not taken into account.

The average loss of life expectancy (at birth) estimated here is slightly less (7.7 months) than the average of all EU citizens (8.6 months) [13]. The rate of premature deaths (76/100 000) is almost the same as the EC study which showed 75/100 000 among EU-25 residents [12].

The total external costs of air pollution estimated here at €150.3 million make up 2.9% of the Tallinn GDP (in 2005). This is somewhat smaller compared to findings from Russia 2.6–6.5% [14] and Beijing 6.55% [38], which are of course much more polluted. But compared to the 1.5% for Europe (WHO assessment), it is slightly higher [13]. The main reason for that might be quite high decrease of life expectancy per premature death case.

The use of HIA as an assessment tool is based on assumptions that local situations and social factors are similar to the reference conditions used in the epidemiological studies from which the exposure-response coefficients are derived. The design of epidemiological studies for long-term effects of air pollution is complicated, especially in small or average size towns where air quality and social patterns vary. Thus, the design used in the current study would be most applicable where resources are limited.

## Conclusion

To some extent, all the citizens of Tallinn are affected by poor air quality. Even though the levels on particulates are not large, still the negative health effects appear. Altogether, 296 premature deaths per year and 3859 YLL, an average loss of 7.7 months life expectancy and 275 hospital admissions due to air pollution make particle pollution a significant environmental health issue in Tallinn. People suffering from chronic diseases should be informed about the air quality in different regions, so that they could avoid these areas. Efforts should be directed to improve the situations in the more polluted sections.

The methodology we used helped to assess the health impacts of air pollution in a town with a sparse monitoring network but where dispersion modeling was available. Sectioning the city for analysis and using GIS techniques helped to improve the accuracy of the impact estimations and helped improve the usefulness of the assessment. It means that these kinds of studies are needed in areas with average pollution levels as well as those with major pollution problems.

## Abbreviations

AirQ: Air Quality Health Impact Assessment Tool; COMEAP: Committee on the Medical Effects of Air Pollutants; EC: European Commission; EHIF: Estonian Health Insurance Fund; EU: European Union; EU-25: European Union with 10 new member states that joined it 2004; GDP: Gross Domestic Product; GIS: Geographic Information Systems; HIA: Health impact assessment; PM_2.5_: fine particles (particles with diameter less than 2.5 μm); PM_10_: particulate matter (particles with diameter less than 10 μm); SVL: Statistical Value of Life; VOLY: Value of Life Year; WHO: World Health Organisation; YLL: Years of Life Lost.

## Competing interests

The authors declare that they have no competing interests.

## Authors' contributions

HO and BF developed the overall concept of current HIA methodology. ET conducted dispersion modeling. TL made economic evaluation and determined baseline health data. TT made GIS designs. MK and VK improved pollution emission database. EM contributed to general health impact background analysis. KK contributed to the interpretation of the analysis results and their applicability in urban risk regulation and HO performed most of the analyses and drafted the manuscript. All authors have read and approved the final manuscript.
